# In memory of Tracey Bretag: a collection of tributes

**DOI:** 10.1007/s40979-020-00066-2

**Published:** 2020-12-30

**Authors:** Sarah Elaine Eaton, Helen Titchener, Ann Rogerson, Saadia Mahmud, Felicity Prentice, Guy Curtis, Katherine Seaton, Rebecca Awdry, Teresa (Teddi) Fishman, Irene Glendinning, Thomas Lancaster, Erica J. Morris, Stella-Maris Orim, Gill Rowell, Michael Draper, Phil Newton, Tomáš Foltýnek, Sabiha Shala, Dukagjin Leka, Zeenath Reza Khan, Jean Guerrero-Dib, Ide Bagus Siaputra, Brian Martin, Robert Crotty

**Affiliations:** Werklund School of Education, University of Calgary, Calgary, Alberta Canada

In this editorial we share our individual experiences of meeting and working with Dr. Tracey Bretag, co-founder and Editor-in-Chief of the *International Journal for Educational Integrity (IJEI)*. Tracey co-founded the journal with Helen Titchener, who worked alongside Tracey as Co-Editor for a number of issues before stepping away. In late 2019, Tracey invited me to join her as Co-Editor-in-Chief, starting in January, 2020. She knew she was ill when she invited me and made it clear that working alongside her during her illness was meant carrying on with the editorship after she was no longer able to do so. Although Tracey book-ended her connection to the journal with Co-Editors, throughout she remained its constant and exemplary leader, steering the journal to successively higher acclaim throughout the past 15 years.

As contributors to this editorial, we share their personal and professional reflections about what working with Tracey has meant to us. Each contributor had a unique and special relationship with Tracey and *IJEI*. One of my tasks was to receive and compile the contributions. I made an editorial decision about how to order the contributions. I have book-ended the editorial with the contributions of Tracey’s Australian compatriots, starting with Helen Titchener’s, as one of the original co-founders of the journal. Next come the contributions of Tracey’s contemporaries in Australia, those who are actively engaged in professional life. Then, I have grouped the contributions by country: United States, United Kingdom, Czechia, Kosovo, UAE, Indonesia, and Mexico. There ordering of the countries is not in any way meant to represent a hierarchy or a preference, and nor is it meant to signal any kind of political statement. We, the educational integrity community the world over, are a global network of colleagues, due in no small part to Tracey’s work. The ordering of the contributions is almost certainly imperfect, and I am grateful for my fellow contributors for their graciousness and generosity. Every individual who has shared the reflections in this collaborative editorial has done so during the middle of the COVID-19 global pandemic, and at a time when we face unrelenting professional demands. The voice of every contributor matters and the ordering of the contributions was no easy task. Having said that, I intentionally ensured that the Australians have the last word, so to speak, as we conclude with the voices of two academic elders, professors *emeriti*, who have supported the journal as members of the editorial board, and Tracey’s work for many years.

Although many individuals have contributed to this editorial, we recognize that we are not alone in being inspired by Tracey. For every person who has shared their reflections in this collective editorial, there are likely at least a hundred others with similar sentiments and have their own personal connections to Tracey. We recognize that our words cannot begin to represent the worldwide impact Tracey had through her work and her vision.

And now on to my own reflection…

My first connection with Tracey was through *IJEI.* The manuscript I had submitted for consideration was one of the first pieces I’d taken the lead on after finally securing a full-time tenure track position, after 22 years of being a sessional lecturer (otherwise known as a precariously-employed academic). The piece was a literature review about the research conducted in Canada on academic integrity. Through the revision process Tracey encouraged me to be bold, to go beyond a literature review, and set the stage for a research agenda in Canada on educational integrity, and to conclude with an evidence-informed call to action. I was inspired by her energy, her clarity of thought, and her passion for the work. I made the revisions and the piece was published (Eaton and Edino 2018).

From there, we stayed in touch and she became a mentor, colleague, and friend, all in one. She joined us as they keynote speaker for the inaugural Canadian Symposium on Academic Integrity at the University of Calgary in April, 2019. She felt unwell during the symposium, but none of us knew at the time, including her, how serious it was. As it turned out, her trip to Canada was one of her last journeys abroad. We are so grateful to have had the opportunity to learn from her in person during her visit.

I have not known Tracey as long as some of my fellow contributors and colleagues. She had an astounding ability to change your life within moments of talking with her. Each time we connected, wisdom, ideas, and inspiration flowed from her as unlimited gifts she gave just by being herself.

Though this collection of tributes, you will see that we grieve individually and collectively, and our heartbreak echoes across continents. Tracey not only *contributed* to our field, she *catalysed* it. Through these reflections we remember her, we celebrate her, and most of all, we honour her.

Sarah Elaine Eaton.

(Co-)Editor-in-Chief, International Journal for Educational Integrity.

University of Calgary, Canada.

## Reflections from Helen Titchener

I remember the day I met Tracey Bretag. It was 18 years ago in a seminar room at UniSA and the occasion was a presentation to introduce a brand new product to university staff. That product was Turnitin®. Tracey had recently begun her Doctor of Education studies and was passionate about pedagogical approaches to addressing plagiarism. I was at the beginning of my own PhD journey, and was passionate to lay bare the depth and breadth of the cheating behaviours of students.

We happened to sit next to each other that day and, just as was the case with so many others that Tracey met at conferences and meetings over the following years, we became confederates and friends. Life-long friends.

Within a fortnight, we had started what was later described by a professor of mine as a ‘cottage industry’. We were young academics, but not young in years and both of us were stubborn and tenacious. So, we did things that all young people should be roundly encouraged to do - we went and knocked on the highest doors and sold our dreams to people who held the ability to make them come true.

We owed much to Professor Alan Bundy, the University Librarian. Alan was acting Pro Vice-Chancellor (Academic) in 2002. We told him that we wanted to grow an international community of academic integrity scholarship and described for him the emerging work in the rest of the world. He listened!

Alan lent us money (seed funding) and introduced us to the people who quickly became the inaugural committee of the Asia Pacific Forum on Educational Integrity. Within a year, the first conference, ‘Plagiarism and Other Perplexities’, was organised and held: We welcomed keynotes from the UK and the US and 200 participants from 12 countries. I was buoyed along by Tracey’s unending enthusiasm and energy and so, doctoral studies, jobs, and family responsibilities notwithstanding, we researched ways to establish a journal. We announced the founding of the *International Journal for Educational Integrity* in the closing ceremony of the conference and 2 years later the first edition was published.

When I had to walk away from my involvement in 2006, Tracey did what she always did, and stepped up to the challenge of doing it herself. Over the following decade and a half, Tracey sat next to countless academics, scholars, and students and never failed to give her time and energy to work with them.

In so doing, she turned our cottage industry into the rigorous, internationally acclaimed, academic endeavour that it is today.

On a final note, it was Tracey who argued for the term ‘*Educational*’ rather than ‘*Academic*’ when referring to integrity, and she was right. The integrity of our students does not begin when they step into university; it is established much earlier and we, as scholars, should extend our research and efforts into those areas.

I am proud to have worked beside Tracey in those earliest of days. We became irrepressible accomplices and life-long friends. Life-long friends in a life that was too short.

(This reflection is based on my contribution to Tracey’s memorial at the University of South Australia on 26 October 2020.)

Helen Titchener.

James Cook University, Australia.

## Tracey Bretag: reflections from Ann Rogerson

A colleague encouraged me to write a conference paper in 2009 to document some work and results related to the use of Turnitin®. Little did I know that my first conference paper (presented at the Asia Pacific Forum for Educational Integrity Conference in Wollongong, Australia) would lead to me meeting Tracey Bretag for the first time, and that this fortuitous meeting would result in a change to my research and career trajectory. This meeting also led to my first publication in the *IJEI*, where I was later honoured to join the Editorial Board. Tracey saw somethings in me that I had not seen in myself – an interest in academic and educational integrity, and the confidence to join the growing chorus of voices wanting to bring the issue out in the open, instead of pretending that the cheating and breaches of integrity did not exist. The *IJEI* is critical to this work, and I am proud to be so closely associated with it.

Many similar stories have been repeated by those fortunate enough to have known Tracey. She recognised in people the sparks of interest in academic integrity and encouraged us to connect, share learnings (appropriately acknowledged of course!) and pursue investigations into issues to encourage learning and quality teaching. Founding the *IJEI*, Tracey could nurture the community while providing an outlet for the talents of those she drew into the academic integrity interest sphere. Through the *IJEI*, she always encouraged individual insights and observations knowing that a range of perspectives was essential to developing a broader and better informed viewpoint on the breadth and extent of cheating behaviours, and how to pursue educative approaches to encourage personal integrity and learning.

Tracey’s passion and efforts highlighted that open discussions and research into the issues around student academic misconduct and contract cheating were essential to drive change in practices, policy and understanding. She also knew that through the open access approach of the *IJEI* that information and research about educational and academic integrity would be available to a wider audience regardless of location. The community of practice saw her bringing together like-minded individuals to work on editorial boards such as *IJEI*, scientific panels, conferences, books, research articles, training programs and presentations in Australia and around the world.

As a role model she demonstrated how academic and educational integrity was worth exploring and researching, and modelled how our responses could contribute to student learning, societies and academic careers. The recognition of her guidance and work around the world is astonishing – but a testimony to her vision, vigour, passion, enthusiasm, drive, approachability, warmth, mentoring, encouragement and support. We are all richer for having known and being influenced by her, but saddened that she was lost to her family and the world too soon. Tracey created a momentum in the area influencing thought, process, policy and governance related to academic and educational integrity that we commit to continuing as an ongoing legacy to her leadership, collegiality and friendship.

Ann Rogerson.

Assoc. Professor Ann Rogerson SFHEA.

Associate Dean (Education).

Faculty of Business and Law, University of Wollongong (Australia), Australia.

Chair UOW Academic Integrity Advisory Group.

## Reflections from Saadia Mahmud

Friends and colleagues, Tracey and I shared a lot of very memorable moments in our journey together as researchers. I first met Tracey in 2006, and we worked on the research on the very contentious issue of self-plagiarism. I remember both of us going back and forth on how to define self-plagiarism. In one of the last pieces she wrote, Tracey mentioned us coming up with the definition of self-plagiarism. From there, through the years, whether it was how to use Turnitin® as an educative tool or how to have a more educative focus on academic integrity policy, or a more humane approach to breaches of academic integrity, we approached the issue with a sense of the holistic and nuanced nature of the problem.

We had some very interesting conversations around the issues. I recall Tracey’s absolute commitment to getting the *IJEI* published, her hunt for reviewers (I was one) and her excitement at receiving interesting research. I worked with her as a project officer on the Academic Integrity Standards project (2010–2012) and Exemplary Academic Integrity project (2013–2014). We identified the five core elements of an exemplary academic integrity policy based on a review of all the policies in the Australian Higher Education sector and the literature. The paper led by Tracey was published in the *IJEI* in 2011. One of my last roles with Tracey was at the Office for Academic Integrity and I recall some of the innovative things we did to reach students on the International Day against Contract Cheating – instant scratchies, snap chat filters and petting Tracey’s dog, Peanut. The fun part was, we got to interact with students, and that was really Tracey’s gift. She knew her topic and was very passionate about it, but at the same time she had great compassion both for students and educators – we struggle as well with how to approach this issue. I remember Tracey used to say that ‘Perfection is the enemy of the very very best’. She wanted the *IJEI* to have continuity and longevity into the future. I witnessed her excitement when Springer took the *IJEI* on as publishers and finally she had the support she needed to publish her beloved journal.

I am grateful for all the years we worked together and for the opportunity to be part of the academic and research integrity story. The last project that Tracey did – the Contract Cheating and Assessment Design project – I had gone away from higher education for a couple of years, but was fortunate enough to come back and be part of that project analysing the largest data set on the topic. Tracey’s lifetime of work on integrity has set Australia on its own track from the rest of the world. I know there are researchers all over the world that are working on this issue, but Tracey’s leadership on this has been phenomenal. Tracey was a dedicated advocate for integrity and a visionary who shall be sorely missed and forever remembered.

Dr. Saadia Mahmud (formerly Saadia Carapiet).

Online Facilitator.

Australian Institute of Business, Australia.

NB: Some of the text is the wording of my video tribute to Tracey for the memorial held at the University of South Australia on 26 October 2020.

## Reflections from Felicity Prentice

A chatty phone call with Tracey led to me revealing an amusing and illuminating story about some students who had used an online paraphrasing tool. In the original essay, in a statement regarding CAT Scans, *angles* had been misspelled as *angels*. The version of the essay spun through the paraphrasing tool rendered this as *Blessed Messengers*. Tracey immediately suggested that this anecdote was the basis for an article in the *IJEI*. For goodness sake, I had never written a peer reviewed article and my PhD candidature was not even confirmed!

Five months later the article was published. It was those 5 months that revealed to me the warmth, depth and extraordinary power that was Tracey. She challenged, encouraged and sustained me through the process. Each word, every sentence, the concepts and contradictions were constructed through her expert guidance and supported by her wisdom and enthusiasm.

Tracey gave me the courage to join the academic community of authors and has illuminated the path forward.

Felicity Prentice.

Lecturer.

Health Sciences.

La Trobe College Australia.

## Reflections from Guy Curtis

I have had two experiences of submitting papers to *IJEI* under the Editorship of Dr. Tracey Bretag, both of which were memorable for how positive they were. I submitted my first article to *IJEI* as a corresponding author in 2008. An abiding memory for me from this submission was that I found the feedback and direction from Tracey, as the Editor, very helpful and supportive. After acceptance of the paper, I recall writing to Tracey to say “thank you” for her help and encouragement in getting the paper in shape for publication, with a statement to the effect that she was the most helpful Editor I had dealt with on a journal article submission. This was among the first half-dozen journal articles in my career; more than 30 papers later, I have not been similarly moved to provide spontaneous positive feedback to an Editor.

In 2010/11, I submitted my second paper to *IJEI*. On receiving the reviews, I remember Tracey, as Editor, apologizing for delays that resulted from the fact that she was being treated for, or recovering from, breast cancer. I recall replying immediately saying that ill health was nothing to apologize for and that the resulting delays were completely understandable. Still, this reply from Tracey showed how important the operation of the journal was to her. Unlike my first submission, my second paper required substantial revision, and I remember Tracey again being clear in her guidance regarding how to address reviewers’ feedback and improve the paper.

As an occasional reviewer for *IJEI*, I found that Tracey was a superb communicator and her enthusiasm for the journal was evident even over email. She clearly knew reviewers’ relevant expertise and expressed her appreciation for our help.

Last year I had the privilege of working with Tracey on her final large project, development and delivery of Australia-wide academic integrity workshops and an academic integrity toolkit for the Australian higher education sector. Through this project, I got to experience Tracey’s enthusiasm, support, high professional standards, and goodwill in person. Tracey was the authentically kind, helpful, and supportive person in real-life as I had experienced as a journal contributor many years before. Tracey’s passing is a terrible loss to her family, friends, colleagues, students, and the community of academic integrity scholars.

Guy Curtis.

Senior Lecturer in Applied Psychology.

The University of Western Australia, Australia.

## Reflections from Katherine Seaton

There are some disciplines and tasks which have for a long time been in the integrity “too hard” basket, non-text disciplines, and in particular mathematics with its proofs and calculations. Tracey had a wide vision for educational integrity, and wanted no discipline sidelined or ignored. She encouraged me to break the silence around mathematics, being only too aware of it because of the overview of the whole educational integrity landscape that editorship of *IJEI* gave her. I will also always remember her compassion for the female students in engineering who report more often giving their work to others, than receiving work from others, while male students report the opposite. Tracey had a rare gift for the big picture and the details, and for distinguishing the offender from the offence.

Katherine Seaton. Associate Professor Katherine Seaton FAustMS, SFHEADepartment of Mathematics and Statistics.

LaTrobe University, Australia.

## Reflections from Rebecca Awdry

I cannot remember when I first met Tracey, but would think that it was around 2012 at the Plagiarism Conference in Newcastle, possibly the year before or after. That was my introduction into the circuit of plagiarism and integrity conferences around the world. I often met Tracey at these events and got to discuss differences between country approaches (myself then being based in the UK), over a coffee or a glass of wine at the networking events. At the time I had completed my master’s thesis on the topic of plagiarism, with a focus in outsourcing. Tracey suggested that I try to turn one of my papers into a journal article, something I’d not yet thought of. She offered to put it in *IJEI* and put me in touch with someone who could co-author with me. She was always very encouraging of new researchers in this field, and given my professional roles for some years were in this area, it seemed a logical step to make for me to move into some form of academia through publishing outcomes of my studies. Having my first article published in *IJEI* was great, and a confidence booster as well!

Following the publication, I ended up moving to Australia, meaning that I would bump into Tracey at national as well as international events. In 2015 I decided to take my interest in this area further, and commenced my PhD on the topic. One of my supervisors at the time was the co-founder of the *IJEI*, Dr. Helen Marsden (now Helen Titchener), so another connection to the journal! Tracey always took the time to talk to me about my research and how I was progressing. I remember being sat in the sun talking at length over a cold drink in Adelaide one hot and sticky afternoon, about the best ways to get information from students to open up and talk about their cheating behaviours. It is thanks to Tracey’s encouragement over 10 years ago that I find myself still writing and researching now.

Tracey was an inspiring advocate for integrity, may she rest in peace.

Rebecca Awdry.

Deakin University, Australia.

## Reflections from Teddi Fishman

I’m not sure if there was a disagreement the very first time I met Tracey, but if not, it was shortly thereafter. Finding and exploring our differences was one of the great joys of our friendship. We disagreed about policies, procedures, and language. We once stayed up hours after a conference’s conclusion, drinking wine and arguing passionately about plagiarism detection/text-matching software. I would give the world to be able to have that argument again.

Despite being ideological allies and self-identified trans-pacific sisters, we argued—in the scholarly sense—constantly. (That’s how it is with sisters.) What made all of our disagreements useful, generative, and often delightful was that regardless of how passionately Tracey believed in her position, she believed just as passionately in testing her assumptions and convictions, which is one of the many reasons she was the perfect person to edit a journal on the subject of educational integrity.

Many of our arguments started the same way: One of us would say something she thought was uncontroversial, and the other would shake their head like a dog shakes off water and say, “What?” and the game was on. The original speaker would state her case and be instantly rebutted. We’d line up our evidence and the debate would rage. After much explaining, nodding, head-shaking and brow-furrowing, the conversation would nearly always turn toward the same place: Talking about how we could test the ideas against each other and find out the truth. The arguments ended in research design plans and hugs.

Tracey possessed the ideal but nearly non-existent combination of passionately held beliefs and openness to conflicting positions that made *her* journal—and I did think of it that way, as her journal —both rigorous and ground-breaking. As was the case in our arguments, no idea was sacrosanct or immune from challenge, but also, no idea was too unconventional to be explored. Just like with our arguments, even though she was a giant in the field of academic integrity, she was willing to consider the possibility that views she had long held might not always hold sway.

Our last argument wasn’t much of an argument at all. We were at an international function dinner, sitting beside each other and chatting as if our life depended on it. We’d finished eating and Tracey reached over and rearranged the silverware on my plate. The look on my face was enough of a question for her to explain that she did it to indicate that I was finished. I said, “Oh, is that how they do it here?” and she said, “This (pointing to her arrangement of the cutlery) means you are finished.” I shared with her that I’m never sure when I’m travelling, because even in the US, we have two different sets of table etiquette rules; I assumed everywhere was different. Tracey told me that she’d always assumed that Americans just didn’t have that kind of table etiquette because (her words) we “aren’t as stuffy as the commonwealth countries.” Then we compared different customs and habits, and gasped at our own misconceptions and assumptions until we were exhausted and laughing about how, after all these years, there was still so much we had to learn.

That was Tracey in a nutshell. She would move the silverware on your plate, and that would launch an exploration of cultural competency and customs. Her endless curiosity, openness and willingness to “go there” made her an ideal editor and an adored and irreplaceable friend.

Teresa (Teddi) Fishman.

Former Director, International Center for Academic Integrity.

United States.

## Memories of Tracey as leader, mentor, friend and colleague

It is with great sadness to have to use the past tense when I talk about Tracey Bretag. She had so much energy and was full of the joys of life. Her presence would illuminate any room, large or small. She was, and still remains, a great inspiration to many people, me included, both in a personal and professional capacity.

It is almost impossible to read anything about academic integrity without coming across Tracey’s name, as a researcher, editor or author. She was, quite rightly, seen as a leading authority in this field, initiating some of the most innovative and influential research conducted anywhere in the world. Her research and publications, completed together with many prestigious colleagues, will continue to be of relevance for many years to come.

I’m personally greatly indebted to Tracey for helping me to develop my skills as a researcher and writer and for encouraging and supporting the first research that I led, 10 years ago, into plagiarism policies. I set out the details of how I first came across Tracey in my article within the Festschrift we put together for the well-deserved ENAI Lifetime Achievement Award, which was presented to Tracey at the (virtual) conference in Dubai in April 2020 (European Network for Academic Integrity (ENAI) 2020), so will not repeat the same narrative here. However, it is worth repeating how much I enjoyed her company and how much fun and energy she brought to any occasion.

Tracey preferred the term *educational integrity* rather than *academic integrity*. We should use this term more, because it encompasses all educational levels. Researchers in this field are increasingly becoming aware of the need for young people to understand about integrity before they go to university, as it applies both to their education and personal lives. Gaining such knowledge and skills throughout their education will prepare young people either for working life or transition to higher education. This is an area where more research and development are needed.

Through the efforts of Tracey, together with researchers she worked with in Australia and elsewhere, there is far more information available today about threats to integrity and ways to challenge and reduce those threats. Tracey’s research helped us all appreciate how commercial contract cheating services are undermining standards in education globally.

The best tribute to Tracey would be evidence of success with the “arms race” against corruption and malpractice in education. The most serious issue facing us today is commercial contract cheating in all its forms, including “essay” mills, degree mills, fake universities, including associated extortion and bribery. This is a global problem therefore we must continue to work together as a community of researchers and activists to find a global solution. To achieve success in this difficult endeavour, we must involve a range of interested parties, especially students.

It is now our responsibility, as an international community of researchers in this field, to build on what has already been achieved by Tracey and others. We must continue to make progress in rooting out cheating in education, in whatever form it occurs.

Dr. Irene Glendinning.

Coventry University, United Kingdom.

## Reflections from Thomas Lancaster

People often assume that, since Tracey and I both started working on academic integrity research in the early 2000s, we’d known each other for a long time. We actually existed in separate circles. While my focus was on educational research and improving pedagogical practice within the UK, Tracey was making an impact on the world stage. Along with Helen Marsden (now Helen Titchener), she launched the *International Journal for Educational Integrity* in 2005. The journal punched beyond its weight from the very first issue, securing contributions from world leading academic integrity researchers of the day, including Don McCabe and Jude Carroll. The journal later became part of the Springer Nature portfolio.

The first time I remember communicating with Tracey was in 2014 as a contributor to the *Handbook for Academic Integrity* she was editing (Bretag 2016). The *Handbook* became and remains one of the great reference sources in the field. Soon after, Tracey invited me to join the Editorial Board of the *International Journal for Educational Integrity*. When Tracey led Australia’s Contract Cheating and Assessment Design project, I was excited to form part of the associated Reference Group. Tracey subsequently became the Lead Advisor for Epigeum’s Academic Integrity training programme and I felt honoured to gain her stamp of approval and become the programme’s only non-Australasian author.

I finally met Tracey in person at the Plagiarism Across Europe and Beyond conference in Brno, Czechia in 2017. Tracey delivered an evidence-informed keynote presentation on contract cheating and proved to be just as friendly and welcoming as I’d expected. Every time we met subsequently, Tracey always greeted me as an old friend. I fully cherish the discussions we had on academic integrity but I will always regret that my path didn’t cross with Tracey’s earlier.

Tracey’s legacy is often seen in her publications, her journal, her edited books and in her advice and guidance to the sector. A strength of Tracey I greatly admired is how she found opportunities to involve so many people from the academic integrity community in her activities. When the tributes for Tracey arrived, I was astounded to see just how many people she’d inspired. To me, the academic integrity community we have today is where we can best see Tracey’s real legacy.

Dr. Thomas Lancaster.

Imperial College London, United Kingdom.

## The impact of Tracey Bretag’s work

The work of Tracey Bretag has had, and will continue to have, a profound impact on the field of academic integrity and long-lasting implications for ensuring academic standards in higher education. Tracey Bretag understood the importance of interdisciplinarity in advancing our understanding of academic integrity issues, and not only embodied and promoted the values of educational integrity, including honesty, trust and fairness, but exemplified inclusive and collegial collaborative approaches through her leadership.

In 2009 to 2011, I led the Academic Integrity Service for the Higher Education Academy and Jisc in the United Kingdom, producing national guidance and leading forums on academic integrity practice and policy for universities (Morris et al. 2010; Morris and Carroll 2011). This initiative built on the highly regarded ‘holistic approach’ for institutions to address student plagiarism, collusion and contract cheating (Macdonald & Carroll, 2006) and a growing research literature that emphasised educational responses to these issues (Hrasky and Kronenberg 2011; Morris, 2016). I began working with Tracey Bretag in 2012, as she had been in touch with me, enthusiastic and complimentary about the academic integrity resources I had produced. She also evaluated such recommendations for practice in the light of her work in Australia on developing effective academic integrity policy (Bretag et al. 2011). I was so fortunate to work closely with Tracey Bretag and colleagues on the Exemplary Academic Integrity Project (2012–2013): she instigated and led a productive Roundtable event, which involved experts and practitioners from a range of higher education institutions, to develop a framework for enacting academic integrity policy. The reach of this work was extended through a national speaking tour in Australia. I am still struck by the ingenuity of the methodology for this project, in which a supportive and collaborative approach enabled evidence-informed reflection and discussion so that recommendations, tutorials, media assets and a toolkit for policy and practice were brought together as a web resource for higher education staff and students.

Tracey Bretag’s work was marked by rigour, underpinned by theory and evidence. She ensured that the serious issue of contract cheating was fully considered in a special collection of the *International Journal for Educational Integrity* through a range of timely perspectives and investigations (Bretag 2017–2018). Tracey Bretag was astute in questioning established good practice guidance on how assessment design can mitigate the possibility of student plagiarism and contract cheating. The research project she led on contract cheating and assessment design was comprehensive, in-depth and extensive: the issue of students’ sharing behaviours and the outsourcing of assessments was unpacked and explored through both staff and student perspectives, with vital evidence-informed guidance produced for institutions and the higher education sector (Bretag et al. 2019; Bretag et al. 2018; Harper et al. 2018). Bretag and Harper (2017) emphasised the need for a systemic approach to address the issue of contract cheating, which has implications for the development of institutional strategy and policy relevant to academic integrity; enhancing teaching, learning and assessment practices; and working with students (Morris 2018).

Tracey Bretag’s academic leadership and thoughtful mentoring of many in the higher education community has been so positive and far-reaching. She has fostered understanding and significantly advanced the field of academic integrity. I will miss Tracey so very much.

Dr. Erica J. Morris.

Principal Fellow of the Higher Education Academy.

Academic Associate (Advance HE).

## Reflections from Stella-Maris Orim

While carrying out my doctoral studies, I was pointed to the comprehensive work on academic integrity carried out by Tracey and her team in Australia. My initial reaction was that of ‘*awe’*. I wondered if there was anything else to add to the body of knowledge in this area of research!

Then in 2012, I attended the 5th International Plagiarism Conference, on 16–18 July 2012, in Newcastle upon Tyne as a session chair and panellist. At the opening ceremony I saw Tracey for the first time, and I was amazed at her passion for this research area as she presented a keynote. I then went on to find her during one of the breaks; introduced myself and talked to her about my research on academic integrity, focusing on student plagiarism and Institutional response. The aura around her was so friendly, welcoming and enthusiastic that I found it difficult to be around Tracey without being infected by her passion - indeed, there were no dull moments around her.

This initial meeting lead to several others as her work contributed immensely to the shaping of my research. Following my meeting with Tracey in Newcastle upon Tyne and discussion of my research with her, I was invited to submit a paper to the *International Journal for Educational Integrity* (IJEI). Tracey was not one to compromise on quality but would rather provide the guidance to achieve a higher standard in the articles the *IJEI* published. This peer review process was rigorous with very detailed feedback and I was quite excited when my paper was published in the *IJEI*.

After this work, Tracey invited me to contribute a book chapter to the *Handbook of Academic Integrity* (Bretag 2016). I was thrilled about this opportunity to showcase my work on the Nigerian Higher Education Institutions. We continued to collaborate over the years, and this led to the invitation to join the Editorial Board of *IJEI* in 2016. I was really delighted that I was entrusted with the opportunity to promote the journal to my colleagues and peers and assist the editor in decision making over issues of the quality of the submitted manuscripts.

We communicated via emails and met severally in other International Plagiarism Conferences and every meeting with Tracey was very memorable as she was very interested in what I was working on and very willing to provide her support. We discussed the possibility of her and Irene Glendinning accompanying me to Nigeria to facilitate Academic Integrity workshops for Nigerian Higher Institution Lecturers – she was always so willing to give her time and share her knowledge.

Tracey has indeed impacted my journey into Academic Integrity research in several positive ways and I really do miss her – Rest in Peace.

Stella-Maris Orim.

Ass. Prof. (SF HEA CMgr CMI).

Course Director, MSc. Management of Information Systems and Technology (MIST).

Faculty of Engineering, Environment & Computing.

School of Computing, Electronics and Mathematics, Coventry University, United Kingdom.

## Reflections from Gill Rowell

Tracey provided keynotes at the International Integrity & Plagiarism Conference which ran in the UK from 2004 to 2014, in both 2012 & 2014. She thrilled delegates with her passionate and research-led presentations and words of wisdom, winning her many great friends in the UK and beyond. I will never forget the iceberg image from her 2012 keynote, showing the unconscious cultural values of integrity which lie beneath the surface!

During this time, I worked with Tracey and Teddi Fishman, then Director of the International Center for Academic Integrity, to establish an alliance of our three integrity conferences. Namely the ICAI annual conference, the Asia Pacific Conference on Educational Integrity and the International Integrity & Plagiarism Conference, as part of a shared commitment to ensuring that conversations on integrity should resonate with, and empower a global community, a vision that clearly was also fundamental to the *International Journal for Educational Integrity*.

Tracey generously invited authors and presenters from the 2012 conference to submit their work to the *International Journal for Educational Integrity*, then published via the UNiSA Open Journal System. The accepted submissions then became part of the final issue for 2012, a year Tracey describes as “very busy year for academic integrity” in her editorial, with “numerous conferences, symposiums and research projects taking place around the globe” (Bretag 2012) of which the 5th International Plagiarism Conference was but one.

Later Turnitin® provided an educational grant for a collection of articles on the rise of contract cheating in higher education, published in the journal in 2017/18. Several years on, the journal had come of age, now under the management of Springer Nature, but still with Tracey’s unwavering editorial vision. During those initial online editorial meetings, often at wholly antisocial times for Tracey in the Southern Hemisphere her passion for the topic and generosity of spirit shone through as ever, and for me, from a personal point of view, it was a great joy to be working alongside her once again.

Tracey has left a formidable legacy behind, with her vision shaping and influencing a whole new generation of educators for whom the values of educational integrity are now second nature. Her spirit and passion lives on in the teachings of those educators and the students they in turn inspire.

Gill Rowell.

Education Manager, Turnitin.

Academic Chair, International Integrity & Plagiarism Conference.

United Kingdom.

## Reflections from Michael Draper and Phil Newton

We have known Tracey for a number of years and Tracey’s passing is a profound loss both personally and professionally.

Tracey was instrumental in advocating and supporting a legal approach to contract cheating which directly led to our collaboration in this area and served as a platform for a range of research and initiatives undertaken by us in Europe not least with the UK Quality Assurance Agency and the ETINED platform of the Council of Europe. Tracey’s influence was global.

Yes, Tracey was a giant in the world of academic integrity but on a personal level she was an absolute joy and the epitome of kindness. Tracey would above all listen and freely share her views with great humour, encouragement and grace.

My last meeting with Tracey was virtual when we delivered keynotes in Vienna 2019 although we had been due to meet up physically at a Conference in Lithuania.

The email that I and others received sums up everything you need to know about Tracey and the care and concern that she had for others:

*“Dear Colleagues,*

*I am so sorry I won’t be at the conference next week – I’m sure it’s going to be fabulous as always!*

*The good news is that my lovely PhD student, Felicity Prentice, will be there and presenting the findings from a paper she published last year in the IJEI. Felicity is in the first year of her candidate but has already read nearly everything ever published on academic integrity – so she probably feels like she knows you all already!*

*I wanted you to have her email address in case you’re trying to find her (I’ve included all three email addresses just to make sure!). She’s also on Facebook if you want to send her a message via Messenger. Please make her welcome at her very first academic integrity conference. I know you’ll have lots to chat about!*

*Enjoy the conference, and know that I’ll be thinking of you all.*

*With warmest regards,*

*Tracey.”*

Professor Phil Newton.

Director Learning and Teaching.

Medical School.

Swansea University, United Kingdom.

Professor Michael Draper PFHEA RLAUKAT.

Director Swansea Academy Inclusivity and Learner Success.

Dean Regulations and Student Cases.

Swansea University, United Kingdom.

## Reflections from Tomáš Foltýnek

I met Tracey for the first time at the conference Plagiarism Across Europe and Beyond in 2013. At that conference 2 years later, the idea of the European Network for Academic Integrity (ENAI) emerged. Since 2017, the conferences have been annual and provide a platform for sharing research results and experience and good practice examples. Tracey was a keynote speaker at almost all of them. We always appreciated her encouraging speeches, valuable feedback to other contributions, and enthusiasm she spread during informal talks and meetings.

Tracey helped us a lot with establishing ENAI. She gladly shared her experience and constructively criticized our ideas and our work. It was a pleasure and a huge benefit to have her on the Advisory board. Without her, ENAI wouldn’t achieve what it is.

It was also the *International Journal for Educational Integrity*, which, thanks to Tracey, provided a venue for the ENAI team’s publications. It started with a special issue in 2013, continued with the publication of selected conference papers in the following years, and resulted in a strong partnership between ENAI and *IJEI*. The aim of this partnership is clear: To share results of the academic integrity research, which allows any higher education institution to adopt good practice from elsewhere and build a culture of academic integrity.

This editorial is too short to enumerate all Tracey’s contributions to ENAI and its community. We all valued Tracey as a colleague, expert, and friend. That is why ENAI appreciated Tracey’s efforts by a Lifetime achievement award earlier this year. Together with the award, ENAI published a Festschrift for Tracey Bretag (ENAI, 2020). In ten chapters, members of the European academic integrity community describe what Tracey meant for them. In their contributions, Tracey is called as “*champion*”, “*absolute legend of the academic integrity world*”, “*very effective critical friend*”, “*prominent figure*”, “*the most selfless, honest and passionate persons you get the honour to meet*”, “*fairy godmother*”, “*family friend*”, “*bundle of energy, bubbling over with great ideas and good stories*”, “*inspirational friend*”, or “*super-leader in the promotion of academic integrity*.” There is no doubt that all these superlatives are true. And this is how we will always remember professor Tracey Bretag.

Tomáš Foltýnek, President of the ENAI Board.

Czechia.

## Tracey Bretag: Memorial essay on friendship

The issue of academic integrity was the preoccupation and main field of research and expertise of Prof. Tracey Bretag. It was she, respectful and professional academician Prof. Tracey Bretag, who became our academic integrity icon. Academic integrity in higher education was and continues to be one of the most challenging aspects for the academic community. Prof. Tracey Bretag gives a lot to this field as a creator of knowledge and as author of many scientific papers in the field of academic integration in education, and in particular in higher education.

The *Handbook of Academic Integrity* (Bretag 2016) is the most precious gift that Prof. Tracey Bretag gave to us 2 years ago on the occasion of her participation at the first Scientific International Conference on Justice and Art held in Kosovo, and after her extraordinary presentation on academic integrity in higher education, as a keynote speaker. The first meeting with our academic integrity giant was in May 2018 during the Conference on Ethics, Transparency and Integrity in Higher Education, where we presented on the state of academic integrity in our institution of higher education. Following that presentation, we were honoured to be invited by Prof. Tracey Bretag to publish in the prestigious *International Journal for Educational Integrity*, a paper entitled “Plagiarism in Kosovo: a case study of two public universities.” It was such an extraordinary experience and privilege to work with Prof. Tracey Bretag in this capacity. Such cooperation certainly continued this year, but this time she left us. Her death saddens us immensely, while your academic wealth for us and future generations remain our treasury.

The scientific and academic world of academic integrity is the feature attributed to her name by all of us today. Humanism, its academic values and its scientific works, are our condolences for her untimely death! Always in our minds and hearts!

Prof. Assoc. Dr. Sabiha Shala, University of Haxhi Zeka, Peja, Kosovo.

Prof. Assoc. Dr. Dukagjin Leka, University of Kadri Zeka, Gjilan, Kosovo.

## Honouring you, Tracey

In the span of 2 min, Tracey Bretag changed the course of my career and life.

In a stroke of serendipity, I was invited to join an executive meeting by a friend who was a friend of a friend who brought Tracey to visit our campus. I was never meant to be a part of that meeting, a mere Instructor at the time. But my friend, who was at the time Director of Institutional Effectiveness and knew of my work in academic integrity said this may be of interest to me.

I was on the brink of changing my PhD thesis topic after 4 years of gruelling, back-breaking work. But it felt like I was fighting a fight that I wouldn’t win, working on a topic that no one cared about. After the meeting, in the corridor outside the elevator, Tracey found out I was planning to change my topic. When I told Tracey, she put her hand on my shoulder, looked me in the eye and said, “I care and I am with you”.

Tracey held my hand as I organized the first International Conference on Academic Integrity – Middle East Chapter. She introduced me to Dr. Teddi Fishman, then-Director of ICAI. When it looked like the conference wasn’t getting much traction, she began inviting colleagues to submit papers and introduced me to the *International Journal of Educational Integrity*. Pledging to accept winning papers for possible consideration and promising to gift two copies of the *Handbook of Academic Integrity* (Bretag 2016), Tracey suddenly made the conference sought-after. We received and ultimately had 20 academic paper presentations and three workshops attended by 60 delegates both national and international!

Tracey pulled me in and taught me to be a reviewer too! Although being a reviewer for *IJEI* wasn’t my first time; however, having Tracey as the Editor-in-Chief meant learning the ropes, etiquettes and coming out of the comfort zone to provide constructive criticism. I learned to provide crucial recommendations on content, context and language. Where before I would have provided a paragraph of review feedback, I now find myself having to work at reducing and cutting down to 2–3 pages!

Tracey didn’t see the work she did with the journal, the reviewers and authors as work. She genuinely cared about academic integrity and all of us. That’s what made us feel like we were a part of a bigger family, pulled from such diverse backgrounds, working side by side towards raising collective voices and concerns over academic misconducts.

Among her umpteen lessons, Tracey taught me to grow resilience and have grit when submitting a paper, to not take feedback personally, to learn from the criticism and strengthen the paper using the comments provided. She said being an academic meant learning to accept rejections and take pride in the acceptances.

Her work and words remain as a constant reminder to me, reflected in her last keynote speech at ENAI’s virtual conference in Dubai “Are we models of integrity in everything we do?” I hope to persevere to answer “yes” in honour of you Tracey, my teacher, confidante, shining star, I still feel your hand on my shoulder. You were a force to reckon with, making people sit up and take notice. I love you and miss you so.

Dr. Zeenath Reza Khan, President, UAE Centre for Academic Integrity.

Assistant Professor and Chair of Academic Integrity Committee, University of Wollongong in Dubai.

UAE

## A pillar for academic integrity

At Universidad de Monterrey (UDEM) we consider appropriate to grant recognition to Dr. Tracey Bretag who was one of the great pillars in promoting a culture of academic integrity worldwide.

We met her in 2014 as a speaker at our 2nd Conference for Academic Integrity. Back then she chaired the International Center for Academic Integrity (ICAI). Then we met her again at the ICAI Conference in Vancouver. Later we accepted her generous offer to spend 3 weeks at UDEM as a visiting professor, then she did the same at the Universidad Católica de Chile.

With the certainty that we are not do it justice, I would like to highlight some of her main achievements and contributions:

- For more than 10 years she focused her research on all aspects of academic integrity.
- She obtained funding from several organizations such as the “Australian Learning and Teaching Council” to carry out different research projects.
- She was former President of the Asia-Pacific Forum on Integrity for Education and President of the ICAI’s Board.
- She was also editor in chief of the *Handbook of Academic Integrity* (Bretag 2016) published by Springer in 2016 and editor and co-founder of the *International Journal for Educational Integrity*. Both works have substantially contributed to the field. Many of us found them as a solid foundation that underpinned our own research projects.
- She published as well the book *A Research Agenda for Academic Integrity* (Bretag 2020) in which expert colleagues from all over the world participated.
- She travelled all over the world defending the values of academic integrity and awakening consciences inside and outside the academic world.

Her impact on the academic world has been enormous and this responds not only to her professionalism and ability, but above all and fundamentally, to her contagious passion and energy with which many of us were captivated.

For many of us at UDEM and in the “Latin American consortium”, she was not only a mentor, she was a respected and admired friend always willing to help and add up.

Thanks Tracey for your hard work, you have left a great legacy. It will take a lot of effort for many of us to fill in your shoes. The task is really challenging but we know it’s the best way to honour your memory.

Jean Guerrero-Dib.

Director of the Center for Integrity and Ethics at the Universidad de Monterrey, Mexico.

ICAI Board Member.

## Tracey is the fundamental values of academic integrity

My preliminary meeting with Tracey began when I attended the 5th International Plagiarism Conference, on 16–18 July 2012, in Newcastle upon Tyne. At that time, for the first time I listened to Tracey’s presentation and was very impressed by her enthusiasm in promoting academic integrity.

The announcement and offer to submit the presented manuscript for further correction and editing before being nominated as one of the articles to be published in *IJEI*, turned the brief meeting into a long-term relationship and collaborations. After receiving feedback and suggestions for improvements, I went through the revision process for several months. Finally, on April 19, 2013, I received good news that my manuscript could be accepted for publication in *IJEI* Volume 9 (2) (Siaputra, 2013).

A few months later, in July 2013, Tracey accompanied 20 students on a visit to Indonesia. On that occasion, I had the rare opportunity to meet in-person and enjoy a warm and insightful discussion with Tracey. At the meeting, I asked for her comment and suggestions regarding the academic integrity (anti-plagiarism) campaign that I have been running since 2012, namely AK.SA.RA. (AcKnowledge, paraphrASe, and integRAte). The results of the discussion and comments provided were materialized in a book chapter in the *Handbook of Academic Integrity* (Bretag 2016), which became one of my monumental works with Tracey. On that occasion, Tracey also explained the differentiation between academic and educational integrity. The term educational is considered more complete and more comprehensive because it can cover all areas of life and involve all members in society, not only the academic community.

Our collaboration continued when I was personally invited to join the editorial board of *IJEI*, on May 19, 2015. I immediately received this honourable opportunity with great enthusiasm and gratitude. My contribution as an editorial board member is not yet worth the amount of trust entrusted to me. One of the assignments given was the publication of Call for papers - Academic integrity in Indonesia: Building on 20 years of progress.

Since 1999, Indonesian government has been aware of various cases of academic misconduct and proclaim the importance of misconduct prevention. In 2010, Indonesian government announced a Ministry of National Education Regulation (MNER) on plagiarism in higher education. Unfortunately, these regulations gave only limited effect on the prevention and eradication of misconduct. The rise of Indonesian’s scientific publication coincided with the increase of ethical violations or academic misconducts, such as contract cheating, manipulation of authorship and citations, multiple submission, or plagiarism.

To perpetuate memories of Tracey and carry on her extraordinary endeavour in the field of academic integrity, I would like to propose an acronym that frames the six fundamental values ​​of academic integrity into her name. I propose to use the word “TRACEY” as the acronym for Trust, Respect, fAirness, Courage, rEsponsibility, and honestY. Hopefully, from now on, every time we talk about academic integrity, we will remember her, because TRACEY is truly the fundamental values of academic integrity.

Ide Bagus Siaputra.

Universitas Surabaya.

Indonesia.

## Reflections from Brian Martin

Tracey and I first made contact on 18 September 2006 when she rang me to talk about integrity matters. We had a lot to talk about. She was organising the Third International Conference on Educational Integrity, to be held in Adelaide. After our phone conversation, she invited me to give a keynote speech.

In December 2007, meeting before the conference, we hit it off immediately. Tracey had an engaging personality; she was genuinely interested in ideas, other people — and educational integrity. Her enthusiasm was infectious. I guessed that she had the same sorts of engaged interactions with others.

By inviting me, Tracey showed her open-mindedness. I was an outsider to the educational integrity area, as a scientist and then a social scientist whose main interests were elsewhere. Furthermore, contrary to the usual focus on student plagiarism, I had written on a range of abuses by academics and other professionals. That suited Tracey fine.

In the years that followed, we kept in touch by phone and email. When contacted by journalists or others about plagiarism and related matters, I often referred them to Tracey as Australia’s leading authority. I think she referred a few people to me.

We shared an interest in self-plagiarism. Using your own text without acknowledging its prior use is widespread in some fields. It’s one of those borderline practices, like hyping your achievements in your curriculum vitae. Despite being deceptive, it is not officially malpractice. Tracey, in collaboration with Saadia Carapiet (now Saadia Mahmud), had creatively used Turnitin to track self-plagiarism among cohorts of Australian academics. It was the most thorough treatment of this topic I knew about.

The *International Journal for Educational Integrity* is one of Tracey’s gifts. It provided a home for a couple of my unusual articles, and I learned a lot serving on the editorial board and reviewing submissions. It is not easy to appreciate the effort required to set up and maintain a journal. Tracey’s wide range of contacts helped make it a success.

I still remember a visit to her beautiful home, where we had stimulating discussions. I also remember her frustrations in her job, including her thwarted attempt at promotion, and her satisfaction at finally overcoming this hurdle. My advice was not to get too upset about her academic rank because research showed that promotion, while initially pleasing, did not make people happier in the long run. Little did I know that only a few years later, with Tracey’s aggressive cancer, academic rank would be the least of her concerns.

Tracey played a crucial role in the educational integrity area that will be impossible for any individual to fill. We can, though, be inspired by her commitment to do what we can in our own ways.

Brian Martin.

Emeritus professor of social sciences.

University of Wollongong, Australia.

## Reflections from Robert Crotty

Most of my own professional life was spent at the University of South Australia.

It was there that, at first sporadically, I met Tracey. I had gravitated to become Dean of Research Degrees in one of the Divisions and I knew of Tracey’s interest in ethics. We exchanged views and she became fascinated with some of the ethical dilemmas I had faced in managing research degrees on the ground. She could not believe some of the wickedness that passed as acceptable practice.

I retired in 2001 as Dean and a couple of years later I was asked to apply for the position of Director of the Ethics Centre of South Australia (ECSA). I was appointed and Tracey and I spent more time together. She would sometimes drop into my home and have a coffee with my wife and myself. I came to know her personal life, which exhibited great sacrifice and commitment, while at the same time she was able to maintain her academic roles. She would also establish the International Journal for Educational Integrity and invited me to the Editorial Board. In so many ways the Journal exhibited the qualities of excellence that Tracey had displayed in her own professional life.

She honoured me by giving several addresses on behalf of the Ethics Centre, always well prepared and often controversial. For example, her ideas on academic self-plagiarism raised eye-brows and caused consternation among some university leaders who wanted to see more production of academic output, without any restrictions. The idea that a staff member might not publish a conference paper, an article and a chapter in a book (or perhaps even a book) without reference to previous incarnations of the substance was something that Tracey deplored and some academic leaders promoted.

Vale, Tracey. What a great woman and parent; what a great academic and ethicist; what a great professional friend.

Robert Crotty.

Emeritus Professor.

University of South Australia, Australia.

Editorial Board Member, *International Journal for Educational Integrity.*

## Data Availability

N/A
